# One Family with Cholestasis: The Twisted Road to the Diagnosis of Pfic 3—Three Case Reports

**DOI:** 10.3390/reports8010033

**Published:** 2025-03-17

**Authors:** Raluca Maria Vlad, Irina Dijmărescu, Ruxandra Dobritoiu, Andreea Moga, Laura Balanescu, Oana Neagu, Daniela Pacurar

**Affiliations:** 1 Department of Paediatrics, “Carol Davila” University of Medicine and Pharmacy, 020021 Bucharest, Romania; raluca.vlad@umfcd.ro (R.M.V.);; 2 “Grigore Alexandrescu” Emergency Children’s Hospital, 011743 Bucharest, Romania

**Keywords:** chronic cholestasis, jaundice, pruritus, metabolic liver disease, genetics, transplantation

## Abstract

**Background and Clinical Significance:** Progressive familial intrahepatic cholestasis (PFIC) refers to a heterogeneous group of autosomal recessive disorders consisting of mutations of hepatocyte transporting-system genes involved in bile formation. The exact prevalence remains unknown but is estimated at 1 in 500.000 for PFIC 3, caused by mutations in the ABCB4 gene. We report three cases of PFIC 3 from the patient’s sister, brother, and cousin, diagnosed in our Pediatric Department in 2022–2023. **Case Presentation:** Case 1: A 10-year-old girl was admitted for jaundice and abdominal pain. She was diagnosed with severely advanced hepatic cirrhosis and massive cholestasis. Genetic testing showed ABCB4 homozygous mutation. She rapidly developed fulminant liver failure, and a living donor liver transplant was performed. Case 2: A 6-year-old brother was previously diagnosed with cholestatic hepatitis of unknown cause back in 2018 and presented with similar features (generalized jaundice, severe pruritus with generalized scratching lesions); symptoms had progressively developed from the first year of life. He also exhibited particular facial features (big forehead, twisted ear lobe, straight nose). He received cadaveric liver transplantation. Case 3: Nephew of first two children, a 3-year-5-month-old boy, was admitted for failure to thrive and a one-year history of jaundice, pruritus, and splenomegaly. He was tested positive for homozygous ABCB4 mutation. He is currently under medical treatment with stable liver function. **Conclusions:** The clinical significance of this particular homozygous variant identified in ABCB4 in our series of cases (c.2534G>T (p.Gly845Val)) was uncertain up to this case report. The present data provide convincing evidence as to the correlation between this mutation and the clinical phenotype of PFIC 3.

## 1. Introduction and Clinical Significance

### 1.1. Background

Progressive familial intrahepatic cholestasis (PFIC) refers to a heterogeneous group of autosomal recessive disorders consisting of mutations of hepatocyte transporting-system genes involved in bile formation. It is responsible for 9–13% of infant cholestasis, with 10–15% of children requiring liver transplantation. The exact prevalence remains unknown but is estimated at 1 in 50.000–100.000 births for PFIC 1, 2 and 1 in 500.000 for PFIC 3 [1,2,3,4,5,6,7,8,9]. Mortality rates range up to 87%; reasons include infections, cerebral/gastrointestinal/splenic bleeding, liver failure, and complications secondary to liver transplantation [10].

Recent molecular studies allowed for the identification of specific genes involved in PFIC, dividing this genetic anomaly into 12 subtypes [1,2]. The most “popular” and well-documented are PFIC 1 (Byler disease or FIC1 deficiency), PFIC 2 (Byler syndrome or BSEP deficiency), and PFIC 3 (MDR3 deficiency) [1,8,9,11].

PFIC phenotype spins around the effects of cholestasis. Thus, children who have objective findings of hyperbilirubinemia are the first to be noticed: scleral or cutaneous jaundice, pruritus, which leads to scratching lesions, irritability, and even cutaneous mutilation; dark urine due to conjugated bilirubin; diarrhea with steatorrhea; vomiting, poor feeding, poor weight gain; epistaxis and bleeding from gums, poor coordination, dry skin, bone weakness/rickets and even nocturnal blindness. Hepatosplenomegaly and altered anthropometric indices like short stature or failure to thrive are encountered [1,3,7,8,12,13,14]

Laboratory findings include high conjugated bilirubin levels. Serum bile salt concentration is 10 to 20 times higher than normal. Serum cholesterol level is usually in the range, whereas high-density lipoprotein level can be normal or low. Alkaline phosphatase and 5-nucleotidase are elevated. Gamma-glutamyl-transpeptidase (GGT) is required when the patient has pruritus and normal bile ducts are observed because it is essential for the differential diagnosis; PFIC 1 and PFIC 2 exhibit normal activity of GGT, whereas in PFIC, three levels are three to ten times higher than normal. Urine samples show elevated bile acid levels, and fecal samples demonstrate high fat levels. If PFIC 2 is suspected, alfa-fetoprotein should be performed to exclude any malignancies [3,13,14,15,16,17].

**Figure 1:**
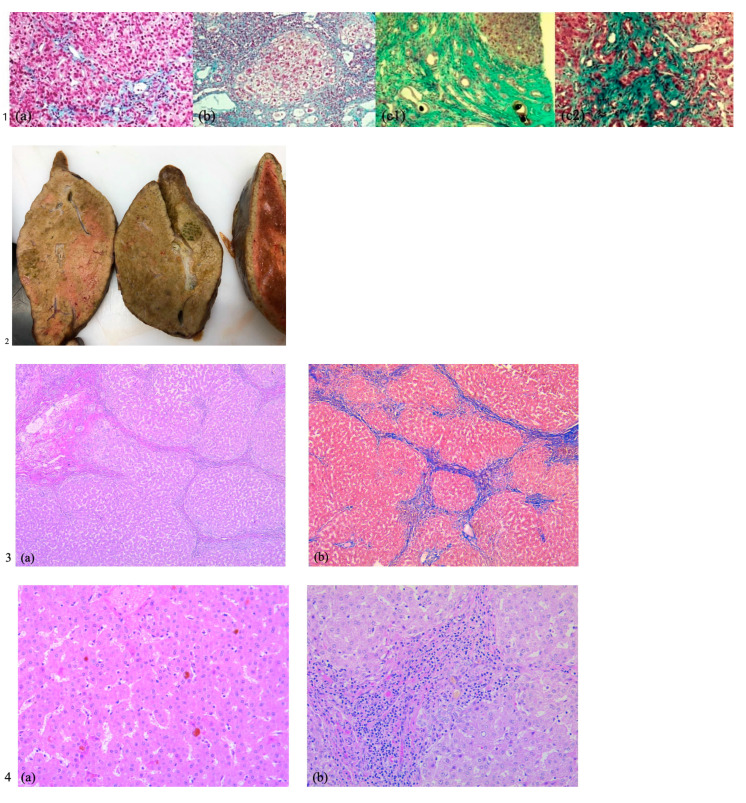
Histology of the PFIC liver. (**1**) Histology images of PFIC 1–3 from the literature [1]. (**1a**) PFIC 1—moderate lobular and portal fibrosis. (**1b**) PFIC 2—hepatocyte necrosis, giant cells, cirrhosis. (**1c1**) PFIC 3—biliary cirrhosis, plugged bile ducts. (**1c2**) PFIC 3—ductular proliferation. (**2**–**4**) PFIC 3 liver in case 2. (**2**) Macroscopic appearance of extracted liver in case 2. (**3**) Liver cirrhosis in case 2. (**3a**) Low power view depicting micronodular cirrhosis with thin fibrous septa with bile ductular proliferation and mild chronic inflammation, HE, 50×. (**3b**) Masson trichrome stain highlights the fibrous septa delineating micronodules in liver cirrhosis, 50×. (**4**) Intrahepatic cholestasis in case 2. (**4a**) Hepatocytes showing cholestasis with pseudo-acinar changes and bile thrombi, HE, 200×. (**4b**): Small portal tract with bile plugs in small canaliculi, ductular reaction, and chronic inflammatory infiltrate, HE, 200×.

A liver biopsy is a required procedure in children with suspected PFIC, as it allows for liver histology and immunostaining to be performed. In PFIC 1, histological findings include hepatocellular and canalicular cholestasis with pseudo-acinar transformation (Figure 1(1a)); giant cell formation and ballooned hepatocytes translate cellular injury. Progression to lobular and portal fibrosis starts early, by the age of two. On electronic microscopy, Byler bile (coarsely granular bile) may be observed in the canalicular spaces. PHIC 2 patients exhibit mainly the same histological traits, but liver architecture is more damaged, with severe lobular and portal fibrosis, as well as inflammation. Necrotizing hepatocytes and giant cells are more perceivable than in subtype 1 (Figure 1(1b)), thus emphasizing that cell injury is more significant in PFIC 2. In PFIC 3, liver biopsy shows true ductular proliferation (Figure 1(1c2)) with mixed inflammatory infiltrate and portal fibrosis; bile ducts are plugged with bile (Figure 1(1c1)). Minimal giant transformation of hepatocytes can be noticed; in time, extensive fibrosis and a typical picture of cirrhosis will develop [1,3,14,18].

Abdominal ultrasonography is the first step imaging tool required to determine the biliary tract anatomy and differentiate PFIC from other causes of cholestasis. Usually, the ultrasound is normal but may reveal a distended gallbladder with gallstones within the biliary tract. Often, computed tomography (CT) and magnetic resonance imaging (MRI/magnetic resonance cholangiopancreatography (MRCP)) alongside isotope scan are performed because they offer a more accurate view of the biliary tree and reveal how well bile is being excreted from the liver. Cholangiography may be necessary to exclude extrahepatic biliary obstruction (such as sclerotic cholangitis) and allow for bile collection for biliary lipid analysis [3,12,13,14].

The management approach for children with PFIC must include experts from the gastroenterology, hepatology, and nutrition departments, pediatric surgeons, as well as psychological counselors in order to find the best path of treatment for these patients. Medical therapy is the first line of treatment in children with all types of PFIC. Objectives are to provide relief from pruritus, improve nutritional status, correct vitamin deficiencies, and approach complications of advanced liver disease such as ascites or variceal bleeding [4,12,19,20,21].

Pruritus is assessed using a five-question scale, which follows the duration of itching, intensity, direction, impact on daily activities, and body distribution. It is called the “5-D Itching Scale” and is a practical tool, especially for children with refractory pruritus [20]. “Frontline treatment” is considered a secondary bile acid called ursodeoxycholic acid (UCDA) at a dosage of 20–30 mg/kg/day. Cholestyramine is a medication that binds to bile acids in the gut and prevents their absorption into the bloodstream. In 2021, the FDA approved an ileal acid bile transporter (IBAT) inhibitor called Bylvay (Odevixibat) for children with PFIC aged 3 months or older. It has proved its efficacy mostly in progressive cholestasis subtypes 1 and 2 [4,12,13,14].

Liver transplant is efficient in children with PFIC and remains the best treatment option, especially for subtype 3 (MDR3 deficiency). In many centers, it is considered a first-line therapy choice [12,13,14,22].

### 1.2. Aim

We aim to report and draw attention to the genetic pedigree of a family with three siblings diagnosed with progressive familial intrahepatic cholestasis type 3 (PFIC 3)—sister, brother, and cousin—diagnosed in the Pediatric Department of “Grigore Alexandrescu” Emergency Children’s Hospital in 2022–2023. The mothers are second-degree cousins; the fathers are second-degree cousins; mothers and fathers are not related to each other.

## 2. Case Presentation

### 2.1. Case 1

A 10-year-old girl was first admitted to our department in June 2022 for scleral jaundice, abdominal pain, mostly left-sided, and poor appetite (a proper history of the disease was difficult to obtain because of illiterate relatives). Symptoms began 4 months prior, and she had never been evaluated by a medical team before. Both her parents were apparently in good health, but her 6-year-old brother was known since 2018 with cholestatic hepatitis of undetermined cause.

On admission, the patient presented with failure to thrive: weight = 23 kg (z-score = −2.03), height = 122 cm (z-score = −2.48), BMI = 15.5 kg/m^2^ (z-score = −0.6), generalized jaundice with scratching lesions, facial angiomas, enlarged liver and spleen. She had dark-brown urine and acholic stools.

Investigations revealed bicytopenia (hemoglobin level = 10.5 g/dL; low platelet count = 134.000/mm^3^), significant liver cytolysis (transaminases x7-10 the upper limit of normal (ULN)), and cholestasis (total bilirubin = 10 mg/dL, predominantly conjugated, high GGT, significantly increased serum bile acids). Infectious causes of acute or chronic hepatitis were promptly ruled out: hepatitis B virus (HBV), hepatitis C virus (HCV), human immunodeficiency virus (HIV), reactivation of Epstein Barr (EBV), and Cytomegalovirus (CMV). Autoimmune liver disorders were highly unlikely (normal ESR, immunoglobulin G and gamma globulins levels, negative anti-LKM1, pANCA, ANA, and ASMA antibodies). Wilson disease and cystic fibrosis were excluded due to normal serum ceruloplasmin, urinary copper, and normal sweat test, respectively. Metabolic storage diseases (Gaucher and Niemann Pick) were also ruled out through genetic testing on dryspot.

Due to family history (brother with chronic cholestatic hepatitis and particular facial features), Alagille syndrome and Progressive familial intrahepatic cholestasis (PFIC) were taken into consideration. Thoracic spine X-ray showed no vertebrae anomalies; cardiac ultrasonography showed no specific malformations, and eye check-up showed no posterior embriotoxon.

Genetic testing (sequence analysis and deletion/duplication testing of the 136 genes listed in the Genes Analyzed section) came out positive for ABCB4 homozygous mutation (c.2534G>T (p.Gly845Val)); the patient is also homozygous for UGT1A1 gene mutation (c.-41-40dup (non-coding)) and heterozygous for NOTCH2 (c.6178C>T (p.Arg2060Cys)), but considering clinical presentation, PFIC type 3 diagnosis was set.

The father was tested with a dry spot for Gaucher and Niemann Pick disease with negative results. Genetic sequencing, WES or otherwise, of the parents was not financially accessible to this family.

**Figure 2:**
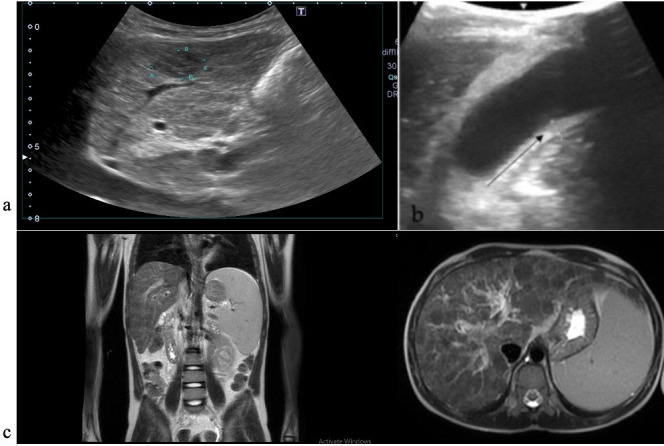
Imagistic findings in case 1. (**a**) Abdominal US—cirrhotic liver nodules. (**b**) Abdominal US—thick-walled gallbladder. (**c**) Abdominal MRI—enlarged liver and spleen.

Abdominal ultrasound found ascites, an enlarged liver with an irregular outline, and multiple nodular elements–hepatic structure alterations compatible with cirrhosis (Figure 2a) and a thick-walled gallbladder (Figure 2b).

Abdominal magnetic resonance described the same cirrhotic aspect of the liver, thick-walled gallbladder with inhomogeneous content, and enlarged spleen (Figure 2c) Upper endoscopy showed esophageal varices with no signs of active bleeding.

PELD score was 18.5 points, meaning 65.5% 1-year waiting list survival and 89.4% 1-year posttransplant survival.

**Figure 3:**
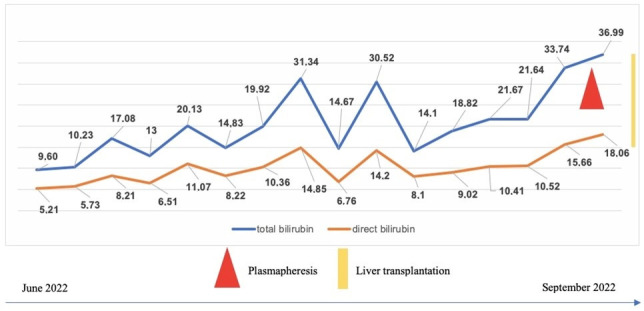
Dynamics of bilirubin levels (mg/dL) in case 1 prior to transplantation.

In the next 2 months, her general condition and jaundice progressively worsened; she developed significant abdominal distension due to rapidly progressing hepatomegaly. Encephalopathy was becoming apparent (altered consciousness, aggressive behavior, reversal of sleep rhythm). Laboratory markers showed progressive thrombocytopenia, severe cholestasis (total bilirubin value of 14 mg/dL, rapidly increasing to 36 mg/dL, predominantly conjugated (Figure 3), significantly altered liver functions (INR = 2.55, hypoproteinemia). Abdominal ultrasound described a medium amount of ascites, and pulmonary X-ray found bilateral pleural effusions.

Partial parenteral nutrition and immediate antibiotic treatment with meropenem were initiated; also, supportive liver medication was given (arginine, plasma infusions, fat-soluble vitamins, beta-blockers, and diuretics), with no improvement of clinical or biological status. Two courses of urgent plasmapheresis did not manage to lower the bilirubin levels.

She rapidly developed liver failure and received life-saving liver transplantation from a related donor (mother), with a good outcome.

### 2.2. Case 2

A 6-year-old boy (brother to case 1), previously diagnosed with cholestatic hepatitis of unknown cause back in 2018 (temporarily lost of follow-up), first presented to our department in June 2022 for generalized jaundice and severe pruritus with generalized scratching lesions. Symptoms had progressively developed since the first year of life. Both his parents were seemingly in good health (disease history was difficult to draw because of illiterate family members).

On admission, the patient presented with stunting: weight = 19 kg (z-score = −0.6), height = 105 cm (z-score = −2), BMI = 17.27 kg/m^2^ (z-score = 1.2), peculiar facial features (triangle-shaped face, large forehead, deep-set eyes, straight nose), xanthomas on hands and feet, generalized jaundice with severe pruritus, and multiple scratching lesions. A heart murmur was discovered during examination, alongside a distended abdomen due to hepatosplenomegaly.

Investigations determined mild anemia, low platelet number, liver enzyme abnormality (transaminases × 2 ULN), cholestasis with a total bilirubin of 3.09 mg/dL predominantly conjugated and high GGT level, and extremely high levels of serum bile acids (×20 times ULN). Infectious causes, autoimmune liver, Wilson disease, cystic fibrosis, and metabolic diseases were ruled out.

**Figure 4:**
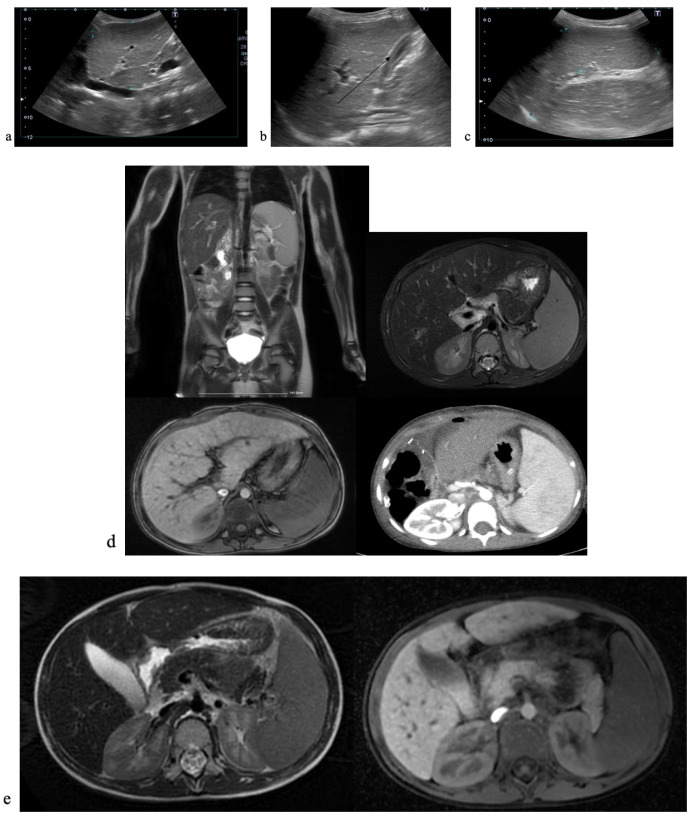
Imagistic findings in case 2. (**a**) Abdominal US—liver nodules. (**b**) Abdominal US—thick-walled, double-layered gallbladder. (**c**) Abdominal US—enlarged spleen. (**d**) Abdominal MRI—enlarged liver and spleen. (**e**) Abdominal MRI—thick-walled, double-layered gallbladder.

Abdominal ultrasound described normal liver size with irregular structure, various nodular elements (Figure 4a), a thick-walled gallbladder (Figure 4b), and an enlarged spleen (Figure 4c).

Abdominal MRI found an enlarged liver with an irregular outline, two nodular structures (probably regenerative type), no bile duct enlargements, and an otherwise normal aspect (Figure 4d), but encountered **a** gallbladder with double-layered walls (Figure 4e). Upper endoscopy showed three esophageal variceal cords with no signs of active bleeding.

Keeping in mind his family history, two main differential diagnosis hypotheses were investigated–Alagille syndrome and progressive familial hepatic cholestasis. Thoracic X-ray showed no vertebrae anomalies; cardiac sonography described normal heart structure, and eye check-up did not reveal posterior embryotoxon. Genetic testing for other inherited diseases associated with intrahepatic cholestasis was not financially available, but considering the older sister had now been labeled as homozygous for ABCB4 mutation, liver biopsy was no longer compulsory for diagnosis, more so because the patient embodied phenotype of PFIC type 3. He was immediately listed for liver transplantation because the father was not compatible as a donor, and the mother had donated the left liver lobe to his sister.

PELD score was 5.6 points, meaning 86.8% 1-year waiting list survival and 93.6% 1-year posttransplant survival.

The patient remained under surveillance at home with medical treatment—ursodeoxycholic acid (UCDA)—to slow down disease progression, fat-soluble vitamin supplementation, hepatocyte preserving medication, and specific alleviating tincture for skin lesions.

In April 2023, the patient underwent surgery for a split adult cadaveric organ grafting with the left liver lobe being transplanted. Regarding the vascular anastomosis, the triangulation method was preferred. The arterial anastomosis was performed via a microscopic approach. An intraoperative ultrasound was performed, and it showed good portal flow and a patent arterial anastomosis with an IR = 0.74 (resistive index). The left biliary duct to the common bile duct anastomosis was performed with biliary stent placement. The stent was removed 1 month after surgery. While the patient’s initial postoperative course was favorable, on postoperative day 3, the control ultrasound could not identify blood flow in the hepatic artery. The patient was taken to the operating room, and an exploratory laparotomy was performed. The liver presented with ischemic areas in segments III and IV-a. Intraoperative ultrasound showed no blood flow in the hepatic artery. A revision of the arterial anastomosis was performed with a good resistive index. The patient’s postoperative outcome was favorable, with an IR of 0.8 in the postoperative ultrasounds.

Pathology aspects of the diseased liver are exhibited in Figure 1 ((1)—literature images, (2–4) histology from case 2).

### 2.3. Case 3

A 3-year-5-month-old boy was first admitted to our Pediatric Department in April 2023 for failure to thrive, one-year generalized jaundice with pruritus, and significant splenomegaly. Both parents and older brother declared no health issues, but we discovered that the patient had two young uncles, who earlier that year, had received liver grafts because of advanced cirrhosis due to progressive familial hepatic cholestasis (both diagnosed in our department—cases 1 and 2).

Upon evaluation, the boy exhibited failure to thrive: weight = 11.5 kg (z-score = −2.6), height = 87 cm (z-score = −2.9), BMI = 15.19 kg/m^2^ (z-score = −0.6). He had generalized jaundice with intense pruritus, second-degree heart murmur, and enlarged liver and spleen. No stool or urine anomalies were noted.

Laboratory investigations showed mild anemia and thrombocytopenia. Liver cytolysis was found (transaminases x2 ULN) alongside cholestasis (total bilirubin 2 mg/dL predominantly conjugated, high GGT) and highly elevated levels of serum bile acids. The coagulation panel was normal.

**Figure 5:**
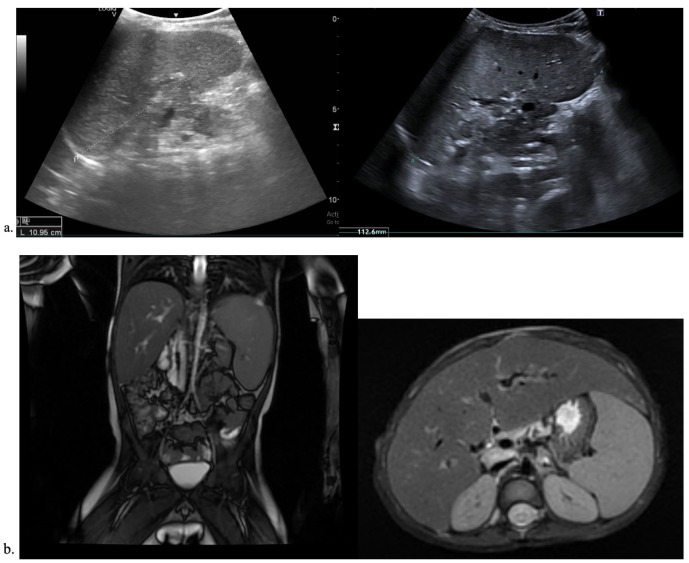
Imaging findings in case 3. (**a**) Abdominal US—hepatosplenomegaly. (**b**) Abdominal MRI—hepatosplenomegaly.

Abdominal ultrasound described an enlarged liver with normal structure and severely enlarged homogeneous spleen. No aspects compatible with liver cirrhosis were encountered, and no alterations of the hepatobiliary tree were found (Figure 5a).

Abdominal MRI described hepatosplenomegaly (right hepatic lobe 110 mm, bipolar spleen axis 114 mm), no expansions of intra or extrahepatic biliary ducts, gallbladder with normal structure, and no ascites (Figure 5b).

Cardiac sonography described normal function of ventricular cavities, no pulmonary hypertension, and intact heart walls.

No esophageal varices were described on upper endoscopy.

Because of the family history (two young uncles diagnosed previously with PFIC type 3), longtime jaundice with pruritus, and severely enlarged spleen with hepatomegaly and cholestasis, based on high conjugated bilirubin values and high level of serum bile acids, we had a reasonable suspicion of PFIC associated with HBV infection.

Genetic testing (sequence analysis using the Blueprint Genetics (BpG) Cholestasis Panel) identified a homozygous missense ABCB4 mutation (c.2534G>T, p(Gly845Val)), as well as his girl cousin who had already been transplanted, so PFIC 3 diagnosis was set.

Parents were tested with dry spots for Gaucher and Niemann Pick disease with negative results. Genetic sequencing, WES or otherwise, was not financially accessible to this family.

He received medical treatment with UCDA, which slightly enhanced the clinical and laboratory statuses (improved jaundice and pruritus, lowered liver cytolysis and cholestasis). Imaging also showed a slightly decreasing size of the liver and spleen. PELD score was 2.2 points, meaning 86.8% for 1-year waiting list survival and 93.6% for 1-year posttransplant survival.

He remains under surveillance and will return for regular laboratory and imaging check-ups. Meanwhile, he will continue medical treatment with UDAC, which, so far, has kept the progression of the liver disease under control. Having a steady liver function, he was not yet listed for liver transplantation.

## 3. Discussion

Genetic analysis is the gold standard for PFIC diagnosis [5,11,23]. Gene analysis requires DNA sequencing of the 27 coding exons and their splice junctions [3]. Nowadays, a resequencing chip that looks for genetic syndromes of cholestasis has been developed and may facilitate diagnosis [24].

PFIC 1 occurs due to mutations within the ATP8B1 gene, which is responsible for producing a protein known as FIC1 (which maintains the amino-phospholipid plasma membrane). This protein is located on the canalicular membranes, cholangiocytes, and cells of the small intestine, which explains its involvement in a variety of functions. That is why a broad spectrum of symptoms exists amongst these mutations [1,3,22,24].

PFIC 2 is caused by mutations in the ABCB11 gene, located on chromosome 2, which encodes for a bile salt export pump (BSEP), a protein responsible for the export of bile salts into the bilious fluid. Mutations led to a buildup of bile salts within the hepatocytes [1,3,6].

PFIC 3 occurs due to a defect in MDR3 (class III multi-drug resistance p-glycoprotein) or a genetic variation in ATP binding cassette subfamily B member 4 gene (ABCB4). Homozygous or compound heterozygous mutations in the ABCB4 gene are considered characteristic variants in PFIC 3 patients. Heterozygous ABCB4 variants result in less severe clinical patterns and partially preserved MDR3 protein function [5]. ABCB4 gene is located on chromosome 7q21 and encodes a translocator protein involved in bile and lipid excretion [2,3,5,8,9,18,21,23,25].

**Figure 6:**
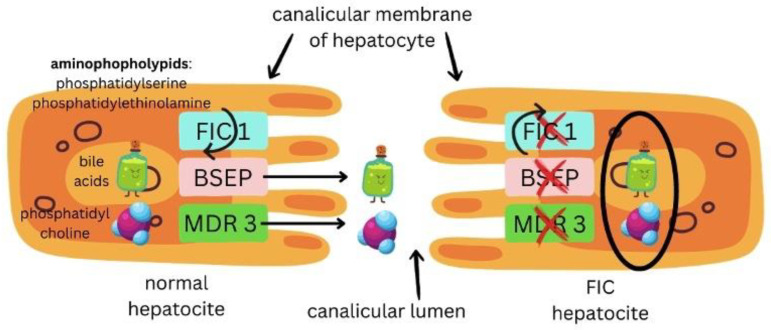
Mechanism of action in PFIC 1, PFIC 2, and PFIC 3.

The mechanism of action of PFIC (all types) is represented in Figure 6.

**Figure 7:**
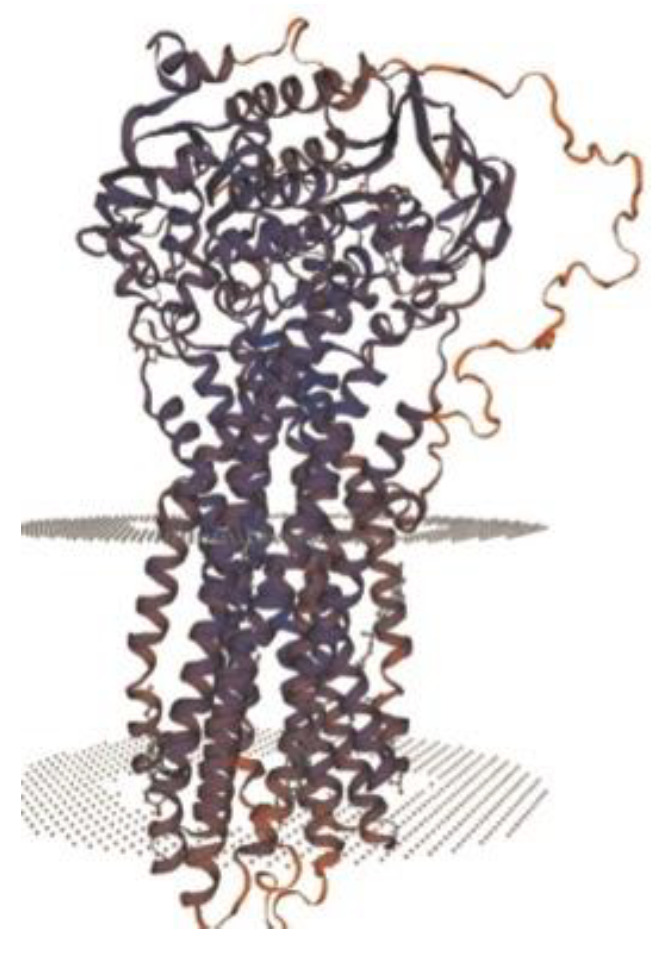
Structure of ABCB4 novel pathogenic variant [6].

Regarding molecular diagnosis, genetic analysis is the gold standard for PFIC 3 diagnosis [6]. Gene analysis requires DNA sequencing of the 27 coding exons and their splice junctions [26]. Nowadays, a resequencing chip that looks for genetic syndromes of cholestasis has been developed and may facilitate diagnosis [27]. In subtype 3, the ABCB4 gene located on chromosome 7 has been confirmed responsible, and approximately 50 mutations have been linked to this form of PFIC [6,28]. ABCB4 protein (Figure 7) is made of two transmembrane domains (TMDs) and two highly conserved nucleotide-binding domains (NBDs). TMDs are in charge of substrate specificity, and NBDs bind and hydrolyze ATP [6]. This protein is actually a phosphatidylcholine (PC) enzyme that transforms PC into bile and emulsified bile salts, thus avoiding injury of the epithelial bile duct cells. Blood samples should be obtained from the child and both parents if possible, more so if they desire a future pregnancy [13].

In our series of three cases, two of the patients (cases 1 and 3) exhibited ABCB4 gene pathogenic mutation. For case 2, complete genetic testing was not financially available (he was only tested for Alagille syndrome, with negative results), but considering the familial aggregation, the patient’s clinical phenotype, and the microscopic aspects of liver biopsy (after transplantation), we may fairly state PFIC 3 diagnosis.

Sequence analysis identified the same homozygous missense variant ABCB4 c.2534G>T, p.(Gly845Val) in both cases genetically tested. This variant is absent in gnomAD, a large reference population database (n > 120,000 exomes and >15,000 genomes) that aims to exclude individuals with severe pediatric disease. This variant affects a moderately conserved amino acid within the ABC transporter, the transmembrane domain of the protein. This variant has been submitted to ClinVar by a clinical testing laboratory (variation in ID 2100150) but has, to the best of our knowledge, not been reported in the medical literature.

The onset of symptoms for PFIC 1 and PFIC 2 is in infancy or early childhood, whereas PFIC 3 can present at any age from infancy to adolescence. The main feature in all types of PFIC is hepatic cholestasis, either in the form of pruritus, jaundice, or both [1,4,6]. Subtype 1 usually debuts with extrahepatic symptoms and is characterized by recurrent episodes of jaundice. In the case of PFIC 2, initial presentation and evolution are more severe; liver failure develops in the first years of life, and hepatocellular carcinoma may complicate the course of this disease. On the other hand, because clinical manifestations of PFIC 3 usually develop later in childhood, patients may present with gastrointestinal bleeding due to cirrhosis and portal hypertension, with or without jaundice. Evolution leads to liver failure, with 50% of patients requiring liver transplantation at a mean age of 7.5 years. No malignancies have been associated with subtype 3 [1,5,6,7,8,9].

**Figure 8:**
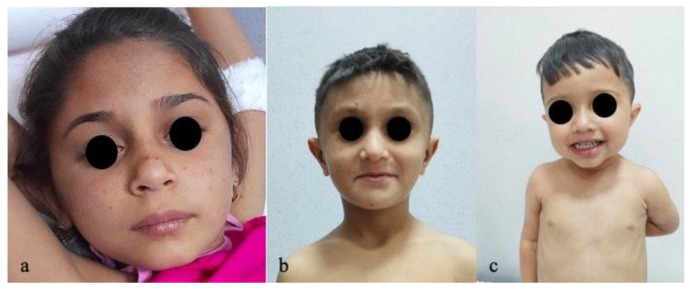
Similar phenotypes with particular facial features. (**a**) Case 1—large forehead, deep-set eyes. (**b**) Case 2—triangle-shaped face, large forehead, deep-set eyes, straight nose. (**c**) Case 3—big forehead, straight nose, twisted ear lobe.

Similar phenotypes in these related patients were obvious on clinical examination in terms of particular facial features (Figure 8) and hepatosplenomegaly.

A liver biopsy is a required procedure in children with suspected PFIC, as it allows for liver histology and immunostaining to be performed. Liver immunostaining is possible due to MDR3 and BSEP antibodies. Mild immunostaining or absent bile ducts favors a gene defect, but normal staining does not necessarily pair with a genetic mutation (a genetic defect may induce function loss with normal synthesis and expression) [3,29].

In PFIC 1, histological findings include hepatocellular and canalicular cholestasis with pseudo-acinar transformation (Figure 1(1a)); giant cell formation and ballooned hepatocytes translate cellular injury. Progression to lobular and portal fibrosis starts early, by the age of two. On electronic microscopy, Byler bile (coarsely granular bile) may be observed in the canalicular spaces [1,3,14,18].

PFIC 2 patients exhibit mainly the same histological traits, but their liver architecture is more damaged, with severe lobular and portal fibrosis, as well as inflammation. Necrotizing hepatocytes and giant cells are more perceivable than in subtype 1 (Figure 1(1b)), thus emphasizing that cell injury is more significant in PFIC 2 [1,3,14,18].

In PFIC 3, histological examination reveals a distinctive pattern closely resembling that of extrahepatic biliary obstruction, characterized by a florid ductular reaction. However, this entity is defined by a patent biliary tree, and these microscopic features align with the MDR3 deficiency, a canalicular transport defect leading to the onset of cholangiopathy [27,30]. Liver biopsy shows true ductular proliferation (Figure 1(1c2)) with mixed inflammatory infiltrate and portal fibrosis; bile ducts are plugged with bile (Figure 1(1c1)). Minimal giant transformation of hepatocytes can be noticed [1,3,14,18,29]. At the onset of PFIC 3, liver histology exhibits portal fibrosis and evident bile ductular proliferation. Most portal tracts showcase interlobular bile ducts, variable degrees of inflammatory infiltrate, and some ducts containing bile plugs and fibrosis. Intralobularly, there is hepatocellular and canalicular cholestasis [31]. Subsequently, there is a progression to marked portal fibrosis and biliary cirrhosis with a micronodular pattern, sometimes accompanied by intraductal cholelithiasis. Notably absent are periductal fibrosis and biliary epithelial injury [32]. These typical changes are found in patient 2’s microscopic pathology, confirming PFIC 3 diagnosis, although genetic testing was not performed.

Differential diagnosis of PFIC includes a large variety of pathologies, from metabolic to endocrine disorders, each one harboring signs and symptoms out of the liver-like area. Clinicians should primarily search for cholestasis evidence, but they must also take into consideration growth pattern, bone status, and hormonal balances because sometimes, the right diagnosis falls somewhere in-between. In order to emphasize the main differential diagnoses, we sorted them alphabetically: A (Aagenaes syndrome, Alagille syndrome, alpha1antitripsin deficiency, arthrogryposis, autoimmune hepatitis), B (benign recurrent intrahepatic cholestasis, bile acid synthetic disorder, biliary atresia), C (choledochal cysts, cystic fibrosis, chole-lithiasis), F (familial Amish hypercholanemia), G (galactosemia), H (hypopituitarism, hypothyroidism), N (North American Indian childhood cholestasis), S (sclerosing cholangitis, sepsis), T (TORCH syndrome, toxic ingestion, tyrosinemia), W (Williams syndrome) [14].

In terms of differential diagnosis, Spraul et al. propose a rather helpful diagram that enables clinicians to have a panoramic view regarding child cholestasis origins [1].

In a patient with a clinical history of cholestasis, as was the case for each of our three patients, after ruling out other main causes such as Alagille syndrome, biliary atresia, cystic fibrosis, sclerosing cholangitis, an alpha1antitripsine deficiency, and extrahepatic bile duct obstruction, PFIC is to be taken into consideration [1,14].

Natural history and complications of subtypes 1, 2, and 3 include portal hypertension, cirrhosis, liver failure, hepatocellular carcinoma, and extrahepatic manifestations. More so, PFIC 1–3 children are at higher risk of developing cholelithiasis and drug-induced cholestasis further in the course of the disease [1,3]. Also, female patients with PFIC who reach adulthood with native liver must continue taking ursodeoxycholic acid (UCDA) during pregnancy to reduce the risk of developing intrahepatic cholestasis of pregnancy (ICP) [1,3]. Children with BSEP deficiency are at high risk of developing hepatobiliary malignancies like hepatocellular carcinoma or cholangiocarcinoma; thus, regular monitoring every 6 months of alpha-fetoprotein and annual abdominal sonography are required for these patients [1,3,33].

Our series of cases clearly illustrates the natural course of a genetic disorder with severe hepatic lesions: three patients with the same disease timely caught in different stages as their respective PELD scores described: 18.5 in case 1 (10-year-old girl), 5.6 in case 2 (her 6-year-old brother), and 2.2 in case 3 (their second-degree 3-year-5-month-old cousin).

Table 1 summarizes the clinical, biochemical, histological, and genetic features of our three cases. The last column contains comparisons between each of our cases and similar ones in the literature.

Agarwal et al. developed a great tool for addressing children with PFIC, starting from dietary and medical treatment options all the way down to liver transplantation [4].

First in line is dietary treatment because poor bile flow means that patients with PFIC are not able to absorb fats and fat-soluble vitamins [12]. Medium-chain triglycerides (MCT) are a special category of easily absorbable fats and represent a solid form of energy for these children. Common foods do not usually contain MCT, but there are some milk formulas and supplements enriched with medium-chain triglycerides. Oral or intravenous supplementation with fat-soluble vitamins (A, D, K, E) is also relevant. Vitamin K 10 mg/week, Vitamin D 3000–5000 UI/day, Vitamin A 2500 UI/day, Vitamin E 50–400 UI/day [4]. Growth impairment, if present, should be approached by setting a nasogastric tube for overnight feedings, thus increasing the protein (3–4 g/kg/day) and energy (180–200 calories/kg/day) intake [4,12].

“Frontline treatment” is considered a secondary bile acid called UCDA at a dosage of 20–30 mg/kg/day. UCDA improves bile flow out of the liver and has numerous benefits, such as reducing bile accumulation in the hepatocellular tissue and reversal of hepatotoxicity, increasing mitochondrial integrity, and decreasing the degree of cholestasis. This medication is particularly useful in MDR3 deficiency [4,12,13,14].

All three of our patients received UDCA with temporary efficacy in case 1 and fairly good results in cases 2 and 3.

Cholestyramine is a medication that binds to bile acids in the gut and prevents their absorption into the bloodstream. A dosage of 200–300 mg/kg/day may reduce itching, although patient feedback varies from case to case. Cholestyramine should not be given at the same time as fat-soluble vitamins because it can stop proper absorption [4,12].

Pruritus had a negative effect on the quality of life of all three patients, but cholestyramine is not available in Romania, so none of them received it.

If, after 4 weeks of therapy with UCDA and Cholestyramine, there is no improvement, alternative medication must be provided. Rifampicin, at a dosage of 5–10 mg/kg/day, is a liver stimulant that upregulates specific detoxification enzymes and may reduce itching in some patients. It has its pitfalls because it can alter liver function tests (must be used with caution in severe liver disease) and can cause urine to turn pink or red [4,12,13,14]. After 8–12 weeks of classical therapy and unresponsive pruritus, other medications may be tried. Phenobarbital, due to its ability to induce CYP enzymes, has long been used to treat newborn hyperbilirubinemia. Naltrexone, at a dosage of 0.25–0.5 mg/kg/day, and Ondansetron, at a dosage of 0.1–0.2 mg/kg/day, can be used for symptomatic relief [4,12,13]. None of our three patients received this medication.

If the severity of pruritus and nutritional status do not improve after 3 months of medical therapy, surgical intervention must be taken into consideration. There are a number of procedures that can be performed in an attempt to reduce the effects of PFIC, but they must be adapted to each patient, and results vary from case to case. The commonest types are internal or external biliary diversion procedures, which prevent enterohepatic recirculation of bile acid salts, thus decreasing serum levels and alleviating itching. External diversion is possible in children who have not yet developed cirrhosis and supposes a conduit connecting the round end of the gallbladder and abdominal skin, creating a permanent communication. Sometimes, a short section of a child’s bowel is used to connect the gallbladder to the surface of the abdomen. Up to 50% of bile flow can be drawn away from enterohepatic recirculation. In addition, other benefits of external diversion are improved hepatocyte function, reduced or reversed progression of disease, increased time until liver transplantation, and prolonged life expectancy. A common complication of this procedure is the collapse of external biliary drainage. Other drawbacks include worsening malabsorption and episodes of cholangitis [12,13,14,20,22,34]. Partially internal biliary drainage is a newer surgical procedure that creates a jejunal connection between the gallbladder and the large intestine, bypassing the ileal reabsorption of bile acids. There is little evidence to back its safety and efficacy, but patients seem to perceive an improvement in their itching status. Effects on hepatocellular function and disease progression should be similar to those induced after external diversion. It can cause diarrhea because more bile enters the colon [12,13,14,22]. An alternative surgical procedure is internal ileal exclusion or ileal bypass, in which the last 15% of the small bowel is bypassed by connecting the proximal ileum to the cecum. This way, the largest site of enterohepatic recirculation of bile acid salts is avoided, providing immediate relief. Downfalls include malabsorption and recurrent cholestasis within the first year after the procedure is conducted [12,13,14].

A great number of patients do not respond to either medical or surgical therapy, developing end-stage liver disease, which will require liver transplantation. Liver grafting has proven its efficacy in children with all types of PFIC and remains, until today, the best treatment option, especially for subtype 3 (MDR3 deficiency). In many centers, it is considered a first-line therapy choice, even in patients with no evidence of end-stage liver disease [12,13,14,22,34].

Cases 1 and 2 progressed to end-stage liver disease and received liver transplantation; case 3 is currently in a steady state in terms of liver function. The timing of liver transplantation was different for case 2 in comparison to his sister because, as there was no family member available as a donor, he received a graft when a compatible deceased donor was found. He presented complications after liver transplantation, including portal thrombosis, which imposed surgical reintervention for de-obstruction. Subsequently, he developed chronic ischemia due to a stenosis of the hepatic artery, highlighted on CT angiography, after identifying persistently high levels of transaminases during follow-up. The evolution after the liver transplant was surprisingly better in case 1, although she received the draft while in critical condition from a possible mutation carrier (the mother) by comparison to case 2, who received a scheduled unrelated deceased donor graft before hepatic failure.

Vascular complications following liver transplant have been reported in up to 22% of cases, more frequently in cases where the graft originated from a living donor and, more often, in younger recipients due to a smaller graft area. These complications can occur in the early period (most related to the hepatic artery and portal vein) or later during evolution. Hepatic artery stenosis has been reported in 4.1% of cases after pediatric liver transplant, less often than hepatic artery thrombosis. Endovascular revascularization is reported to have an 85.7% success rate, in comparison to surgical revascularization, which reaches 100% success rate. The spectrum of clinical symptoms includes minor elevations of liver function tests to acute fulminant failure [35,36,37].

When looking at our two transplanted cases (cases 1 and 2), vascular complications occurred for the younger patient who received the graft from a deceased donor, which is not typical. Portal thrombosis was found shortly after the transplant and was surgically resolved. However, hepatic artery stenosis developed later during follow-up and was identified because of persistently elevated transaminases, even though it is usually reported to be an early-period vascular complication.

## 4. Conclusions

Cholestasis is one of pediatrics’ greatest challenges in terms of diagnosis and management. Whenever a child has chronic cholestasis for an unknown reason or unsatisfactory therapeutic effects, after ruling out infectious and autoimmune causes, clinicians should take into consideration genetic liver disorders.

Genetic testing is becoming an indispensable tool for diagnostic purposes and should be promptly accessed because it provides additional guidance to clinicians about the likely course of the disease. Targeted gene sequencing has proven its value in rapidly and reliably discriminating cholestatic liver disease in children. ABCB4 gene mutations result in progressive familial intrahepatic cholestasis type 3, homozygous or heterozygous status being responsible for the severity of the disease. Not all variants present in a gene cause disease. The clinical significance of this particular homozygous variant identified in ABCB4 in our series of cases (c.2534G>T (p.Gly845Val)) was uncertain up to this case report. The present data provide convincing evidence of the correlation between this mutation and the clinical phenotype of PFIC 3.

Pruritus is the “editor-in-chief” symptom of the storybook, known as cholestasis, which is the most upsetting feature for patients with PFIC. There is a large palette of medications used for relieving itching, such as UCDA, Questran, or Phenobarbital, but in the long term, none has proven efficacy. Surgical procedures help patients in terms of alleviating pruritus and improving nutritional status but have a high risk of complications and recurrence. Liver transplantation is the best and only “curable” choice for children with PFIC 3, prolonging life expectancy and ensuring a better quality of life.

## Figures and Tables

**Table 1:** Clinical, biochemical, histological, and genetic features in our series of cases compared to the literature data.

Our Series Cases	ClinicalFeatures	BiochemicalFeatures	HistologicalFeatures	GeneticFeatures	Similar cases in Literature
Case 1	Symptoms began 4 months prior: - scleral jaundice - abdominal pain, mostly left-sided - poor appetite - facial angiomas - enlarged liver and spleen - dark-brown urine, acholic stools - failure to thrive: weight = 23 kg (z-score = −2.03) height = 122 cm (z-score = −2.48) BMI = 15.5 kg/m^2^ (z-score = −0.6)	Bicytopenia: hemoglobin = 10.5 g/dL, low platelet count = 134.000/mm^3^ Cytolysis: ALT x7 ULN (250 U/L), AST X10 ULN (451 U/L) Cholestasis: total bilirubin = 10 mg/dL, conjugated bilirubin = 5.21 mg/dL, GGT = 176 U/L, significantly increased serum bile acids NEGATIVE auto-antibodies: anti-LKM1, pANCA, ANA, ASMANEGATIVE: HIV, CMV, EBV, HVB, HVCNORMAL viral serologies: serum ceruloplasmin, urinary copper, sweat test	no biopsies	negative for Gaucher, Niemann- PickHomozygous for ABCB4 mutationc.2534G>T Homozygous for UGT1A1 mutationHeterozygous for NOTCH2	Qiao et al. [25]: A 25-year-old female had cryptogenic cirrhosis, splenomegaly, portal hypertension, ascites, chronic cholecystitis, with history of recurrent abnormal hepatic function for 8 years (high GGT and bile acid), jaundice, itching, fatigue Heterozygousmutations in ABCB4 gene: c.2696C > G
Case 2	Symptoms had progressively developed since the first year of life: - generalized jaundice - severe pruritus - generalized scratching lesions - particular facial features (triangle-shaped face, large forehead, deep-set eyes, straight nose) - xanthomas on hands and feet - heart murmur - hepatosplenomegaly - stunting weight = 19 kg (z-score = −0.6), height = 105 cm (z-score = −2), BMI = 17.27 kg/m^2^ (z-score = 1.2)	Mild anemia (hemoglobin = 10.2 g/dL), low platelet count = 166.000/mm^3^ Low grade cytolysis: AST, ALT x2 ULN (138 U/L, 130 U/L) Cholestasis: total bilirubin = 3.09 mg/dL, conjugated bilirubin = 1.71 mg/dL), GGT = 150 U/L, extremely high levels of serum bile acids (x20 ULN) NEGATIVE auto-antibodies: anti-LKM1, pANCA, ANA, ASMA NEGATIVE viral serologies: HIV, CMV, EBV, HVB, HVCNORMAL: serum ceruloplasmin, urinary copper, sweat test	- micronodular cirrhosis - thin fibrous septa with bile ductular proliferation - mild chronic inflammation - hepatocytes with cholestasis, pseudo-acinar changes, and bile thrombi - small portal tract with bile plugs in small canaliculi - ductular reaction (chronic inflammatory infiltrate)	negative for Gaucher, Niemann- Pick Genetic testing for others was not financially available. Considering that older sister was labeled homozygous for ABCB4 mutation and that the patient has PFIC type 3 clinical phenotype, liver biopsy was no longer compulsory for diagnosis.	
Case 3	- one-year generalized jaundice - pruritus - significant splenomegaly - second-degree heart murmur - failure to thrive: weight = 11.5 kg (z-score = −2.6) height = 87 cm (z-score = −2.9) BMI = 15,19 kg/m^2^ (z-score = −0.6)	Mild anemia (hemoglobin = 10.6 g/dL), thrombocytopenia = 148.000/mm^3^ Cytolysis: AST, ALT x2 ULN (97 U/L, 59 U/L) Cholestasis: total bilirubin = 2 mg/dL, conjugated bilirubin = 0.98 mg/dL, GGT = 118 U/L, highly elevated level of serum bile acids NEGATIVE viral serologies: HIV, CMV, EBV, HVCPOSITIVE: HVB antigen, undetectable viremia	no biopsies	negative for Gaucher, Niemann- Pick Homozygous for ABCB4 mutation	Alasmari et al. [18]: 5 years old male patient with history of EBV infection and family history of liver disease (1 uncle with liver transplantation died at 30 years; another uncle died at 11 years, both of unknown causes) Homozygous pathogenic variant c.2906G>A in ABCB4 gene presented with hepatosplenomegaly and cytopeniawithout a history of jaundice or itching.

## Data Availability

The original contributions presented in this study are included in the article. Further inquiries can be directed to the corresponding author.

## References

[1] Davit-Spraul A., Gonzales E., Baussan C., Jacquemin E. (2009). Progressive familial intrahepatic cholestasis. Orphanet J. Rare Dis..

[2] Bai J., Li L., Liu H., Liu S., Bai L., Ning H., Song W., Zou H., Wang X., Chen Y. (2021). A novel compound heterozygous mutation in ABCB4 gene in a pedigree with progressive familial intrahepatic cholestasis 3: A case report. Ann. Transl. Med..

[3] Jacquemin E. (2012). Progressive familial intrahepatic cholestasis. Clin. Res. Hepatol. Gastroenterol..

[4] Agarwal S., Lal B.B., Rawat D., Rastogi A., Bharathy K.G., Alam S. (2016). Progressive Familial Intrahepatic Cholestasis (PFIC) in Indian Children: Clinical Spectrum and Outcome. J. Clin. Exp. Hepatol..

[5] Zhu H., Wang S., Li L., Geng W., Wan X., Hua R., Wang D., Gao P. (2022). Case Report: A rare case of young adult progressive familial intrahepatic cholestasis-type 3 with a novel heterozygous pathogenic variant of ABCB4. Front. Pediatr..

[6] Waisbourd-Zinman O., Surrey L.F., Schwartz A.E., Russo P.A., Wen J. (2017). A Rare BSEP Mutation Associated with a Mild Form of Progressive Familial Intrahepatic Cholestasis Type 2. Ann. Hepatol..

[7] Sun G.R., Burns M. (2015). Progressive Familial Intrahepatic Cholestasis: A Rare Cause of Cirrhosis in Young Adult Patients. Case Rep. Med..

[8] Zhang B.P., Huang Z.H., Dong C. (2019). Biliary atresia combined with progressive familial intrahepatic cholestasis type 3: A case report and review of the literature. Medicine.

[9] Lipiński P., Ciara E., Jurkiewicz D., Płoski R., Wawrzynowicz-Syczewska M., Pawłowska J., Jankowska I. (2021). Progressive familial intrahepatic cholestasis type 3: Report of four clinical cases, novel ABCB4 variants and long-term follow-up. Ann. Hepatol..

[10] Baker A., Kerkar N., Todorova L., Kamath B.M., Houwen R.H. (2019). Systematic review of progressive familial intrahepatic cholestasis. Clin. Res. Hepatol. Gastroenterol..

[11] Zampaglione L., Rougemont A.L., Rubbia-Brandt L., Abramowicz M., Guipponi M., Marchionni E., Valerie M., Goossens N. (2023). Variable Intrafamilial Expression of ABCB4 Disease. ACG Case Rep. J..

[12] (2018). Progressive Familial Intrahepatic Cholestasis (PFIC). Children’s Liver Foundation (Fighting Childhood Liver Disease).

[13] A Siddiqi I., Tadi P. (2024). Progressive Familial Intrahepatic Cholestasis. [Updated 3 July 2023]. StatPearls.

[14] Wehrman A.J., Kennedy M. Progressive Familial Intrahepatic Cholestasis Treatment & Management. https://emedicine.medscape.com/article/932794-treatment#d6.

[15] Constantin A.T., Streata I., Covăcescu M.S., Riza A.L., Roșca I., Delia C., Tudor L.M., Dorobanțu Ș., Dragoș A., Ristea D. (2023). Genetic Testing for Familial Hypercholesterolemia in a Pediatric Group: A Romanian Showcase. Diagnostics.

[16] Constantin A.T., Delia C., Tudor L.M., Rosca I., Irimie A.D., Năstase L., Gherghina I. (2023). Dyslipidemia in Pediatric Patients: A Cross-Sectional Study. Medicina.

[17] Chen H.L., Chang P.S., Hsu H.C., Lee J.H., Ni Y.H., Hsu H.Y., Jeng Y.M., Chang M.H. (2001). Progressive familial intrahepatic cholestasis with high gamma-glutamyltranspeptidase levels in Taiwanese infants: Role of MDR3 gene defect?. Pediatr. Res..

[18] Goubran M., Aderibigbe A., Jacquemin E., Guettier C., Girgis S., Bain V., Mason A.L. (2020). Case report: Progressive familial intrahepatic cholestasis type 3 with compound heterozygous ABCB4 variants diagnosed 15 years after liver transplantation. BMC Med. Genet..

[19] McKiernan P., Bernabeu J.Q., Girard M., Indolfi G., Lurz E., Trivedi P. (2023). Opinion paper on the diagnosis and treatment of progressive familial intrahepatic cholestasis. JHEP Rep..

[20] Elman S., Hynan L.S., Gabriel V., Mayo M.J. (2010). The 5-D itch scale: A new measure of pruritus. Br. J. Dermatol..

[21] Azami M.A., Lahbali O., Lamalmi N., Oukabli M., Bouzidi A.A. (2017). Progressive Familial Intrahepatic Cholestasis Type 3: A Case Report and Literature Review. J. Pediatr. Child. Health Care.

[22] Jankowska I., Pawłowska J., Szymczak M., Ismail H., Broniszczak D., Cielecka-Kuszyk J., Socha P., Jarzębicka D., Czubkowski P. (2021). A Report of 2 Infant Siblings with Progressive Intrahepatic Familial Cholestasis Type 1 and a Novel Homozygous Mutation in the ATP8B1 Gene Treated with Partial External Biliary Diversion and Liver Transplant. Am. J. Case Rep..

[23] Mirza N., Malhotra S., Sibal A. (2020). A Novel Compound Heterozygous Mutation in ABCB4 Gene Leading to Cholelithiasis, Progressive Familial Intrahepatic Cholestasis (Type 3), and Cirrhosis in a Child. J. Child Sci..

[24] Torfenejad P., Geramizadeh B., Haghighat M., Dahghani S.M., Zahmatkeshan M., Honar N., Imanieh M., Malekhosseini S.A. (2016). Progressive Familial Intrahepatic Cholestasis and its Subtypes: The First Report From Iran. Iran. J. Pediatr..

[25] Li G., Lin H., Zhang A., Li J., Wang M., Fang X. (2021). Infantile cholestasis related to ABCB4 gene mutation and cytomegalovirus (CMV) infection: A case report. Dig. Med. Res..

[26] Alasmari B.G., Rayees S., Alomari M., Elzubair L., Hamid Y. (2022). Progressive Familial Intrahepatic Cholestasis Type 3 Homozygous Pathogenic Variant c.2906G>A in the ATP Binding Cassette Subfamily B Member 4 (ABCB4) Gene: A Case Report of an Unusual Presentation. Cureus.

[27] Qiao F., Ren F., Lu W., Yang H., Mo G., Wang S., Liu L., Xu X. (2023). A female of progressive familial intrahepatic cholestasis type 3 caused by heterozygous mutations of ABCB4 gene and her cirrhosis improved after treatment of ursodeoxycholic acid: A case report. BMC Med. Genomics.

[28] Wei G., Cao J., Huang P., An P., Badlani D., Vaid K.A., Zhao S., Wang D.Q., Zhuo J., Yin L. (2021). Synthetic human ABCB4 mRNA therapy rescues severe liver disease phenotype in a BALB/c.Abcb4^-/-^ mouse model of PFIC3. J. Hepatol..

[29] El-Guindi M.A., Sira M.M., Hussein M.H., Ehsan N.A., Elsheikh N.M. (2016). Hepatic immunohistochemistry of bile transporters in progressive familial intrahepatic cholestasis. Ann. Hepatol..

[30] Alsohaibani F.I., Peedikayil M.C., Alfadley A.F., Aboueissa M.K., Abaalkhail F.A., Alqahtani S.A. (2023). Progressive Familial Intrahepatic Cholestasis: A Descriptive Study in a Tertiary Care Center. Int. J. Hepatol..

[31] Morotti R., Suchy F., Magid M. (2011). Progressive Familial Intrahepatic Cholestasis (PFIC) Type 1, 2, and 3: A Review of the Liver Pathology Findings. Semin. Liver Dis..

[32] Vitale G., Gitto S., Vukotic R., Raimondi F., Andreone P. (2019). Familial intrahepatic cholestasis: New and wide perspectives. Dig. Liver Dis..

[33] Namgoong J.M., Hwang S., Kim D.Y., Ahn C.S., Kwon H., Ha S., Kim K.M., Oh S.H. (2022). Living donor liver transplantation in an infant patient with progressive familial intrahepatic cholestasis along with hepatocellular carcinoma: A case report. Korean J. Transplant..

[34] Hüpper M.N., Pichler J., Huber W.D., Heilos A., Schaup R., Metzelder M., Langer S. (2023). Surgical versus Medical Management of Progressive Familial Intrahepatic Cholestasis-Case Compilation and Review of the Literature. Children.

[35] Li W., Bokkers R.P., Dierckx R.A., Verkade H.J., Sanders D.H., de Kleine R., van der Doef H.P. (2024). Treatment strategies for hepatic artery complications after pediatric liver transplantation: A systematic review. Liver Transpl..

[36] Ozturk M., Dag N., Sigirci A., Yilmaz S. (2021). Evaluation of Early and Late Complications of Pediatric Liver Transplantation with Multi-slice Computed Tomography: A High-Volume Transplant Single-Center Study. Turk. J. Gastroenterol..

[37] Karjoo M., Kiani M.A., Sarveazad A., Saeidi M. (2017). Short and Long Term Complications after Pediatric Liver Transplantation: A Review and Literature. Int. J. Pediatr..

